# Impact of a natural disaster on access to care and biopsychosocial outcomes among Hispanic/Latino cancer survivors

**DOI:** 10.1038/s41598-020-66628-z

**Published:** 2020-06-25

**Authors:** Mary Rodriguez-Rabassa, Ruthmarie Hernandez, Zindie Rodriguez, Claudia B. Colon-Echevarria, Lizette Maldonado, Nelmit Tollinchi, Estefania Torres-Marrero, Adnil Mulero, Daniela Albors, Jaileene Perez-Morales, Idhaliz Flores, Julie Dutil, Heather Jim, Eida M. Castro, Guillermo N. Armaiz-Pena

**Affiliations:** 1 Clinical Psychology Program, School of Behavior and Brain Sciences, Ponce Health Sciences University, Ponce, Puerto Rico; 2 Division of Mental Health, Ponce Research Institute, Ponce, Puerto Rico; 3 Division of Cancer Biology, Ponce Research Institute, Ponce, Puerto Rico; 4 Department of Basic Sciences, Division of Pharmacology, School of Medicine, Ponce Health Sciences University, Ponce, Puerto Rico; 5 Department of Cancer Epidemiology, H. Lee Moffitt Cancer Center and Research Institute, Tampa, Florida United States; 6 Division of Women’s Health, Ponce Research Institute, Ponce, Puerto Rico; 7 Department of Basic Sciences, Division of Microbiology, School of Medicine, Ponce Health Sciences University, Ponce, Puerto Rico; 8 Department of Basic Sciences, Division of Biochemistry, School of Medicine, Ponce Health Sciences University, Ponce, Puerto Rico; 9 Department of Health Outcomes and Behavior, H. Lee Moffitt Cancer Center and Research Institute, Tampa, Florida United States

**Keywords:** Cancer epidemiology, Human behaviour

## Abstract

Cancer is the leading cause of death in Puerto Rico (PR). Hurricane Maria (HM) and its aftermath lead to widespread devastation on the island, including the collapse of the healthcare system. Medically fragile populations, such as cancer survivors, were significantly affected. The goal of this study was to assess the impact of HM on barriers to care, emotional distress, and inflammatory biomarkers among cancer survivors in PR. This exploratory longitudinal study was conducted in health care facilities and community support groups from PR. Cancer survivors (n = 50) and non-cancer participants (n = 50) completed psychosocial questionnaires and provided blood samples that were used to assess inflammatory cytokines levels. Among this cohort, we identified 41 matched cancer survivors/non-cancer participants pairs. Data were analyzed through descriptive, frequencies, correlational, and regression analyses. Cancer survivors that were affected by HM reported increased barriers in accessing medical care, which were directly associated with anxiety, perceived stress, and post-traumatic symptomatology. Moreover, being a cancer survivor, predicted more barriers to receiving health care, especially in the first six weeks after the event, after which the effect was attenuated. Several inflammatory cytokines, such as CD31, BDNF, TFF3, Serpin E-1, VCAM-1, Vitamin D BP, and PDGF-AA, were significantly upregulated in cancer survivors while MMP9 and Osteopontin both had significant positive correlations with barriers to care. HM significantly impacted Puerto Ricans psychosocial well-being. Cancer survivors had significant barriers to care and showed increased serum inflammatory cytokines but did not show differences in anxiety, stress, and post-traumatic symptoms compared to non-cancer participants.

## Introduction

Natural disasters can significantly alter an individual’s daily life and lead to psychological distress, particularly in medically fragile populations such as cancer survivors. Hurricane Maria (HM) made landfall in Puerto Rico (PR) on September 20, 2017, as Category 4 storm, killing an estimated 2,975 people and causing an estimated $90 billion in damages^1–3^. Widespread devastation included loss of power and potable water infrastructure; destruction of buildings, bridges, and roads; lack of telecommunications; and closing of ports and airports^4^. Lack of access to food and clean water was a significant problem for residents of PR^4^. Mudslides rendered many roads in rural areas impassable, limiting relief efforts and access to medical care^4^. The severity and duration of the aftermath caused significant psychological distress in the Puerto Rican population^5^. Suicide rates rose by 29% compared to the year before (2016)^5^. During the subsequent three months after HM made landfall, the government psychosocial helpline received 2.4 times more calls to manage suicidal attempts^5^. At that same time, the Behavioral Risk Factor and Surveillance (BRFS) database documented that Puerto Ricans reported: (1) decreased attendance to routine medical check-up in the last 12 months (from 81% to 77%), (2) increased prevalence of depression (from 17% to 19%), (3) increased smoking rates (from 11% to 12%), and; (4) increased obesity (from 32% to 36%)^6^. The majority of excess deaths after HM were attributed to complications associated with chronic diseases^7^. However, knowledge about psychological distress and biobehavioral factors after a natural disaster, such as HM, in cancer survivors, remains limited.

Cancer survivors suffer from physical adversities and present a higher risk of possible psychological deterioration^8^. Data indicate that 30% to 40% of cancer survivors report symptoms associated with emotional distress, such as adjustment disorder, extreme anxiety, and depressive disorders, as a result of the illness and treatment^9^. Another factor that adds to the psychological distress of a cancer survivor is interruptions of their medical treatment^10^. The lack of access and interruptions of medical treatment may be due to cultural disparities such as high costs of cancer treatments, low income in Latino populations, transportation problems, and poor access for cancer specialists^11^. These disparities can be aggravated due to the appearance of a natural disaster, severely altering the psychological state of cancer survivors and interrupting their medical treatment^12^. Natural disasters might adversely impact social and community structures, creating difficulty for individuals to access various resources, such as food, shelter, medical facilities, and other critical requirements for affected people^13^. Patients with chronic illnesses, such as cancer, are one of the most vulnerable groups after disastrous events, due to greater challenges encountered concerning medical access^13^. Recent studies have shown that cancer survivors exposed to natural disasters, such as hurricanes, results in decreased long-term survival^14,15^. Other studies have documented that traumatic stress can be common and persistent after a hurricane^16–20^ and can be associated with alterations in circulating markers of inflammation (decreased natural killer cell cytotoxicity, higher white blood cell count) in individuals without cancer^19^. Of note, exposure to chronic stress is a risk factor for the onset and morbidity of other physical diseases (cardiovascular diseases, diabetes, asthma, metabolic syndrome) *via* exacerbation of systemic inflammation^21–23^. Also, cancer survivors with depression and anxiety demonstrate altered cytokine profiles^24,25^. The extreme stress that a natural disaster, such as HM, can place on a vulnerable population such as cancer survivors is concerning as it has been reported that chronic stress and depression can accelerate the growth of existing tumors and promote chemoresistance^26–33^.

Planning for short- and long-term patient cancer care requires a holistic approach that considers the impact of psychological distress at the biological level in the context of a natural disaster. Extreme environmental stressors might have a significant effect on cancer survivors and could lead to an increased prevalence of psychological distress, such as chronic stress and depression, that have been associated with disease progression^34^. More importantly, exposure to natural disasters, such as hurricanes, resulted in worse outcomes and decreased long-term survival^14,15^. In light of these data, it is important to determine whether and to what extent psychological and social distress (caused by an extreme environmental stressor) can contribute to altered behavioral states and systemic cytokine levels in cancer survivors. The goal of this study was to assess the impact of HM on barriers to care, emotional distress, and inflammatory biomarkers among cancer survivors in PR.

## Materials and Methods

The study followed the ethical principles from the Declaration of Helsinki and was approved by the Institutional Review Board of the Ponce Medical School Foundation (IRB Approval 080121-IF). We obtained written informed consent from all participants at the time of enrollment.

### Participants

This prospective longitudinal case-control study collected participants’ blood and self-reported measurements. All measurements were obtained at the time of recruitment and every three months for one year following the landfall of HM (September 20, 2017). Participant recruitment started four months (January 2018) after HM made landfall and ended in August 2019. Recruitment was carried out in healthcare facilities in the southern part of PR along with a collaborative network that included the Puerto Rico BioBank, a tertiary hospital in Ponce, PR, and cancer survivor support groups. Inclusion criteria included: age (participants had to be between 21 and 89 years old), living in PR during HM, and stayed on the island for the next three months and the duration of this study follow up. Additionally, participants were seeking or in the process of receiving healthcare services. Interested individuals were excluded if they informed or documented existing major psychiatric or neurologic conditions that were not controlled or prevented them from participating in this study. We screened and evaluated 111 cancer survivors which we recruited the first 50 participants that met the inclusion criteria. Most of the cancer survivors approached that did not participate in this study were on active treatment or moving to other regions (including mainland USA) to seek healthcare. We screened and evaluated 84 participants without a previous or current cancer diagnosis and recruited the first 50 that met the inclusion criteria. The majority of individuals not enrolled had plans to move to other regions (including mainland USA) due to the lack of electricity, water service, and road closures. Among our cohort, we identified 41 matched cancer survivors/non-cancer participants pairs. The two groups were matched by age and sex with no significant differences observed (age *p* = 0.086; sex *p* = 0.464). Matching resulted in a final case-control study of 82 participants (41 cancer survivors and 41 non-cancer participants). Matching cases to control were performed in a blinded manner (1:1 ratio caliper distance = 0.63, without replacement) using the MatchIt R package. These parameters resulted in a better match between cases and controls without compromising excessively the sample size.

### Natural disaster outcomes

The research team developed the Natural Disaster Outcomes Questionnaire, based on their personal experiences with HM, to identify problems suffered by participants in the aftermath of the hurricane. This survey contains 23 Likert-type questions that explore if and to what degree participants were impacted by hurricane-related problems during the previous three months. Total scores range from 0 to 92, with higher scores reflecting higher impact. The “Natural Disaster Outcomes” questionnaire was developed based on a literature review that included reports that assessed the aftermath of natural disasters, such as hurricanes, in different populations^35–37^. Furthermore, our group identified the outcomes reported after HM through the local press that highlighted the lack of electricity and the collapse of communication systems^38,39^. This questionnaire was subjected to a research team review and content validation by individuals that experienced HM and its aftermath. Preliminary analyses of the reliability of the questionnaire that excluded missing values showed excellent internal consistency (α = 0.936). The questionnaire is included as Supplementary Data 1.

### Psychological distress and access to health care

Participants answered a battery of questionnaires that explored psychological distress. Scoring and interpretation of these questionnaires followed the guidelines and procedures reported in the literature. To gather participants’ reports of depression (e.g., little interest or pleasure in doing things), they answered the PHQ-8 questionnaire (α = 0.82)^40,41^. Anxiety symptomatology (e.g., worrying too much about different things) was assessed using the GAD-7 questionnaire (α = 0.89)^42–44^. Participants were asked to complete the Distress Thermometer, which assesses psychological discomfort on an 11-point scale (0 = no distress; 10 = extreme distress)^45^. This scale has shown satisfactory diagnostic accuracy (area under the curve ≥0.82, sensitivity ≥90%, specificity ≥64%, positive predictive value ≥25%, and negative predictive value ≥97% for a selected DT cutoff of (5)^46,47^. Perception of stressful situations (e,g., feeling that he/she was unable to control important things in his/her life) was evaluated with the PSS (α = 0 .82)^48,49^. The PCL-5 (α = 0.96) was used to explore PTSD symptomatology (e.g., repeated, disturbing, and unwanted memories of the stressful experience)^50,51^. We assessed resilience or the ability to bounce back (e.g., It does not take me long to recover from a stressful event) throughout the BRS (α =  0.83)^52,53^. Post-traumatic growth (e.g., I know better that I can handle difficulties) was measured with the PTGI-SF (α = 0 .83)^54,55^. Perception of social support (e.g., If I were sick, I could easily find someone to help me with my daily chores) was also assessed using the ISEL-12 (α = 0.70)^56^. To assess potential barriers that participants faced with access to health care (e.g., Having to wait too many days for an appointment) after the hurricane, we utilized the BCQ (α =  0.95)^57,58^. To better understand the study results, the research team inverted the scoring system so higher scores would now indicate higher barriers.

#### Blood processing, storage and cytokine array analyses

Blood samples were obtained at the time of recruitment and processed within four hours of collection time to isolate serum following standard methods. Serum samples were stored at −80 °C. Serum was analyzed with R & D Proteome Profiler Human XL Cytokine Array kits (Minneapolis, MN) according to manufacturer’s instructions. Arrays were quantified using Quick Spots Tool in Western Vision’s HLImage++ (Version 22.0). Cytokine heatmaps were constructed in RStudio (Version 1.0.153) with the following R packages: RColorBrewer, d3heatmap, and ggplot2. To identify significant differences in cytokine patterns between cancer survivors and controls, volcano plots were constructed using GraphPad Prism (version 8.4.1). Protein-protein interaction network and gene enrichment of differentially expressed cytokines were constructed with STRING online platform (version 11.0).

### Statistical analysis

Variable distributions were assessed with the Kolmogorov-Smirnov test to determine the statistical approach required. With the exception of the PSS Scale, scores for all variables were not normally distributed. We used non-parametric methods to describe the outcomes (e.g., median and interquartile ranks) based on participants’ history of cancer. We used Spearman Rho correlations to explore associations between variables and Mann-Whitney U tests to test group differences for the outcomes, including cytokine levels. We performed quantile regression analyses to predict barriers in access to care by cancer status, age, and time of recruitment after HM made landfall. All statistical analyses were conducted with RStudio. We used the RStudio statistical program with the following packages: nortest, psych, olsrr, qgraph, dplyr, Hmisc, corrplot, tidyselect, ggpubr, gplots, RColorBrewer, d3heatmap, and ggplot2. All tests were 2-sided, and statistical significance was defined as *p* < 0.05. Cytokine expression analyses were performed using raw expression values (arbitrary units) and differences in cytokine values between cancer survivors and non-cancer participants. Cytokine distribution normality was assessed using the Shapiro-Wilk test. We performed Mann-Whitney analyses to identify significant differences in cytokine expression between groups. The statistical significance threshold for cytokine analyses (volcano plot) was established at p < 0.01.

### Data sharing

The data that support the findings of this study are available from the corresponding author upon reasonable request.

## Results

### Study population

Here we studied the biopsychosocial effect of a natural disaster (HM) on 82 Hispanic/Latino participants (41 cancer survivors and 41 non-cancer participants) matched for age and sex. The mean time of recruitment after the hurricane was 7.74 months (range: 4.33–11.27 months). Table 1 shows the sociodemographic characteristics of study participants, while Table 2 shows data obtained from psychosocial questionnaires administered to all participants. In general, the majority of the participants were women (71%). Breast (54%) and prostate (17%) cancers were the most prevalent cancer types among cancer survivors. The age of cancer survivors [mean = 56.1 (standard deviation (SD) = 12.4)] was slightly higher from non-cancer participants (mean = 51.5, SD = 12.5), *p* > 0.05.

**Table 1:** Clinical and demographical measurements from study participants.

Characteristics	Cancer Survivors (n=41)	Non-Cancer Participants (n=41)	*P*-value
Age (mean, range)	56 (25-75)	52 (24-73)	0.086
Sex (male/female)	10/31	13/28	0.464
Race	Hispanic (41/41)	Hispanic (41/41)	
Type of cancer	n (%)		
Breast	22 (54%)		
Prostate	7 (17%)		
Endometrial	3 (4%)		
Cervix	3 (4%)		
Lung	1 (1%)		
Other	5 (12%)

**Table 2:** Psychosocial measurements from study participants.

Assessments	Cancer Survivors			Non-Cancer Participants			*P*-value
	Mean (SD)	Median (IQR)	%	Mean (SD)	Median (IQR)	%	
Natural disaster outcomes	21.22 (16.74)	17.00 (9.00–32.50)	95.1	30.00 (23.83)	26.00 (9.00–43.00)	97.6	0.135
No water	1.17 (1.61)	0.00 (0.00–3.00)	41.5	2.15 (1.68)	2.00 (0.00–4.00)	71.0	0.008*
Food insecurity	0.76 (1.33)	0.00 (0.00–1.00)	41.5	1.44 (1.53)	1.00 (0.00–3.00)	58.5	0.017*
Difficulties accessing roads	0.66 (1.20)	0.00 (0.00–1.00)	26.8	1.32 (1.64)	0.00 (0.00–3.00)	46.3	0.045*
Family insecurity	0.73 (1.05)	0.00 (0.00–1.00)	14.5	1.56 (1.55)	2.00 (0.00–3.00)	43.9	0.003*
Depression symptoms	6.76 (5.52)	6.00 (2.50–9.50)		5.83 (5.70)	4.00 (1.50–9.00)		0.330
Moderate to severe (n, %)	8 (19.5%)			8 (19.5%)			
Anxiety symptoms	5.71 (4.74)	5.00 (2.00–7.00)		5.85 (5.35)	5.00 (1.00–10.50)		0.882
Moderate to severe (n, %)	8 (19.5%)			11 (26.8%)			
Post-traumatic stress symptoms^‡^	15.76 (15.47)	11.00 (5.00–22.00)		14.95 (16.19)	10.00 (2.00–23.00)		0.531
Scores >33 (cut-off) (n, %)	6 (14.6%)			6 (14.6%)			
Emotional distress^†#^	3.85 (3.02)	4.00 (1.00–7.00)		3.92 (3.11)	4.00 (1.00–6.00)		0.965
High levels of distress^†#^ (n, %)	21 (52.5%)			21 (53.8%)			
Perceived stress (PS)^‡^	15.20 (6.95)	16.00 (11.50–20.00)		14.58 (7.16)	15.50 (10.00–18.00)		0.508
Moderate to high PS^‡^ (n, %)	26 (63.4%)			31 (62.5%)			
Resilience	3.56 (0.94)	3.33 (2.83–4.50)		3.39 (0.67)	3.33 (2.92–3.83)		0.515
High resilience (n, %)	12 (29.3%)			7 (17.1%)			
Post-traumatic growth	35.00 (13.87)	41.00 (27.50–45.00)		38.29 (9.48)	40.00 (34.50–45.00)		0.676
Great/very great growth (n, %)	24 (59%)			29 (71%)			
Perceived social support	10.34 (7.46)	9.00 (3.50–16.00)		8.12 (7.16)	6.00 (1.50–14.00)		0.149
Barriers in access to care^†§^	18.11 (18.48)	10.58 (2.88–30.77)	30	12.23 (12.78)	6.73 (1.60–18.43)	17.5	0.225
Skills	9.98 (13.86)	3.13 (0.00–18.75)	14.6	5.72 (10.17)	0.00 (0.00–7.81)	2.4	0.318
Marginalization^‡^	16.13 (19.77)	9.09 (0.00–26.14)	24.4	10.20 (13.57)	4.55 (0.00–17.05)	12.2	0.129
Expectations^§^	23.75 (31.71)	3.57 (0.00–41.96)	30.0	16.03 (22.09)	7.14 (0.00–21.43)	22.0	0.515
Knowledge and beliefs	18.29 (27.47)	6.25 (0–28.13)	24.4	10.98 (18.05)	0.00 (0.00–15.63)	17.1	0.390
Pragmatics^†^	23.58 (20.77)	16.67 (4.17–38.89)	43.9	19.38 (17.52)	12.50 (2.78–33.33)	30	0.383

% expressed as percent of participants that were affected by each variable on the NDO and Barriers in Access to Care questionaires; ^‡^Non-Cancer, n=40; ^†^Non-Cancer, n=39; ^§^Cancer, n=40; ^#^Cancer, n=40, ^*^Significant Differences: p < 0.05.

### Natural disaster outcomes

Participants reported being negatively affected by all items identified in the Natural Disaster Outcomes Questionnaire (Tables 2 and 3). Cancer survivors showed lower median total scores (Mdn = 17.00, IQR = 9.00–32.50) than non-cancer participants (Mdn = 26.00, IQR = 9.00–43.00), but this difference was not significant (*p* = 0.135). Nearly a quarter of cancer survivors (24%) and a third of non-cancer participants (32%) faced the loss of a loved one. The most frequent problems faced by participants from both groups were no electricity service (cancer: 68%; non-cancer: 90%) and difficulties with communication services (cancer: 71%; non-cancer: 88%), but these rates were not significantly different between groups. However, reports on the degree of discomfort caused by natural disaster-related outcomes were usually higher in non-cancer participants than cancer survivors. For example, non-cancer participants reported higher rates of being affected by having no water service, no food, food insecurity, and loss of security for the family (*p* < 0.05).

**Table 3:** Natural Disaster Outcomes Questionnaire responses by item from study participants. Reported as % of participants from each group that were affected by the outcome described by the item.

Item	All	Cancer Survivors (n=41)	Non-Cancer Participants (n=41)	*P*-value
	%	%	%	
No electricity^	79.0	68.3	90.0	0.152
Loss of home	26.8	24.4	29.3	0.580
Loss of a loved one	28.0	24.4	31.7	0.505
No water	56.1	41.5	70.7	0.008*
Lack of food	36.6	26.8	46.3	0.045*
Lack of food security	50.0	41.5	58.5	0.017*
Difficulty accessing treatment	34.1	31.7	36.6	0.294
Difficulty accessing medications#	30.9	30.0	31.7	0.681
Difficulty accessing roads	54.9	46.3	63.4	0.116
Financial issues	54.9	53.7	56.1	0.453
Lack of home security	35.4	26.8	43.9	0.073
Employment lay-off or reduction in labor hours^#^	27.2	17.5	26.6	0.084
Long lines at gas stations	58.5	48.8	68.3	0.278
Trafic jam	65.9	58.5	73.2	0.626
Difficulties with internet	64.6	58.5	70.7	0.487
Difficulties with communications	79.3	70.7	87.8	0.356
Cost of generators acquisition	46.3	41.5	51.2	0.371
Cost of generators maintenance (gas or diesel, oil)	42.7	39.0	46.3	0.295
Lack of personal space	31.7	29.3	34.1	0.408
Lack of social support	26.8	26.8	26.8	0.703
Family separation	31.7	22.0	41.5	0.052
Loss of vehicle	11.0	7.3	14.6	0.289
Loss of security for my children and family	29.3	14.6	43.9	0.003*

^Non-Cancer, n = 40; ^#^Cancer, n = 40; *Significant Differences among cancer survivor group and non-cancer participants (p < 0.05)

### Psychological distress

Cancer survivors showed higher symptomatology in several measures of psychological distress compared to non-cancer participants (Table 2). The median scores of depression symptomatology for cancer survivors were 6.00 (IQR = 2.50–9.50) versus 4.00 (IQR = 1.50–9.00) in non-cancer participants *(p* = 0.33). Analyses of the severity of symptoms showed an equal number of cancer survivors (n = 8, 20%) and non-cancer participants (n = 8, 20%) distributed in the categories of moderate to severe depression. Similarly, post-traumatic symptomatology scores in cancer survivors were slightly higher than non-cancer participants (Mdn = 11.00, IQR = 5.00–22.00 vs. Mdn=10.00, IQR = 2.00–23.00; *p* = 0.53), with an equal number of participants showing increased severity of post-traumatic symptomatology (scores higher than 33) (cancer 15%, non-cancer 15%).

Both groups revealed comparable scores on measures of anxiety symptomatology that were not significantly different (cancer: Mdn=5.00, IQR = 2.00–7.50; non-cancer: Mdn=5.00, IQR = 1.0–10.50), emotional distress (cancer: Mdn=4.00, IQR = 1.00–7.00; non-cancer: Mdn=4.00, IQR = 1.00–6.00), and perceived stress (cancer: Mdn=16.00, IQR = 11.50–20.00; non-cancer: Mdn=15.50, IQR = 10.00–18.00). Scores on resilience (cancer: Mdn=3.33, IQR = 2.83–4.50; non-cancer: Mdn=3.33, IQR = 2.92–3.83), post-traumatic growth (cancer: Mdn=40.00, IQR = 27.50–45.00; non-cancer: Mdn=40.00, IQR = 34.50–45.00) and perception in social support (cancer: Mdn=9.00, IQR = 3.50–16.00; non-cancer: Mdn=6.00, IQR = 1.50–14.00) were also not significantly different.

### Access to health care

Cancer survivors reported higher barriers in access to care compared to non-cancer participants (Table 2). Cancer survivors had higher median scores on the total scale (cancer: Mdn=10.58, IQR = 2.88–30.77 vs non-cancer: Mdn=6.73, IQR = 1.60–18.43) as well as in the Skills subscale (cancer: Mdn=3.13, IQR = 0.00–18.75 vs. non-cancer: Mdn=0.00, IQR = 0.00–7.81), but these differences did not reach statistical significance (*p* > 0.05). The median score on the Marginalization subscale was greater in cancer survivors compared to non-cancer participants (Mdn = 9.09, IQR = 0.00–26.14 vs. Mdn=4.55, IQR = 0.00–17.05, respectively) although not significant (*p* > 0.05).

**Figure 1:**
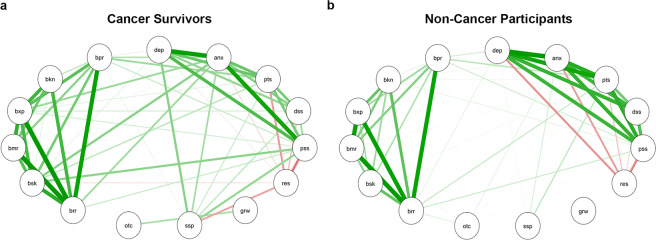
Correlation network of psychosocial assessments in (**a**) cancer survivors and (**b**) non-cancer participants groups. Only significant correlations are shown as determined by qgraph. Green lines: positive correlations; red lines: negative correlations. Line thickness shows the strength of the correlation. All comparisons shown are statistically significant. *P* < 0.05. *brr*: barriers to care – total; *bsk*: barriers to care – skills; *bmr*: barriers to care – marginalization; *bxp*: barriers to care – expectations; *bkn*: barriers to care – knowledge and beliefs; *bpr*: barriers to care – pragmatics; *dep*: depressive symptomatology; *anx*: anxiety symptomatology; *pts*: post-traumatic stress disorder symptoms; *dst*: distress; *prc*: perceived stress; *rsl*: resilience; *grw*: post-traumatic growth; *scs*: perceived social support; *otc*: natural disaster outcomes.

Next, we explored associations between participants’ scores on various psychosocial questionnaires used in this study for each group, using the Spearman’s Rho correlation test. Figure 1 is a visual representation of a correlation network with significant associations (*p* < 0.05) in cancer survivors (A) and non-cancer participants (B), generated using qgraph in RStudio 1.1.463. Both groups displayed a direct correlation between scores of depression, anxiety, post-traumatic symptoms, distress, and perceived stress. Resilience scores were inversely related to these measures in all participants, except for depression, which showed no association. Resilience was also negatively associated with barriers to care (skills subscale), but only in cancer survivors. All measures of barriers in access to care were directly inter-associated in both groups, but reports of such barriers by cancer survivors showed significant positive associations with scores on anxiety, perceived stress, and post-traumatic symptoms. Interestingly, the perceived social support scores in cancer survivors revealed a positive relationship with depression, anxiety, post-traumatic symptoms, distress, and perceived stress. In summary, the psychosocial variables exhibited stronger associations among them in cancer survivors than in non-cancer participants.

We next explored the relationship between being a cancer survivor and barriers in access to care, the primary outcome of this study. Quantile regression analyses confirmed a significant association between barriers in access to care and cancer status (Supplementary Table 1). Being a cancer survivor was associated with increased barriers to access to care scores (Supplementary Table 1), while an impact magnitude of cancer history on barriers in access to care was amplified and reached statistical significance in the upper quantiles of the distribution. Specifically, being a cancer survivor increased barriers in access to care score by 15.64 points for the 90^th^ quantile of the distribution (CI:1.53–23.49, *p* = 0.03, model adjusted for age and sex). We then assessed whether this relationship was maintained independently of the time of recruitment. Interestingly, the difference in barriers to access to care between cancer survivors and non-cancer participants was more marked early after the event and attenuated over time (Supplementary Figure 1).

### Serum cytokine analyses

**Figure 2:**
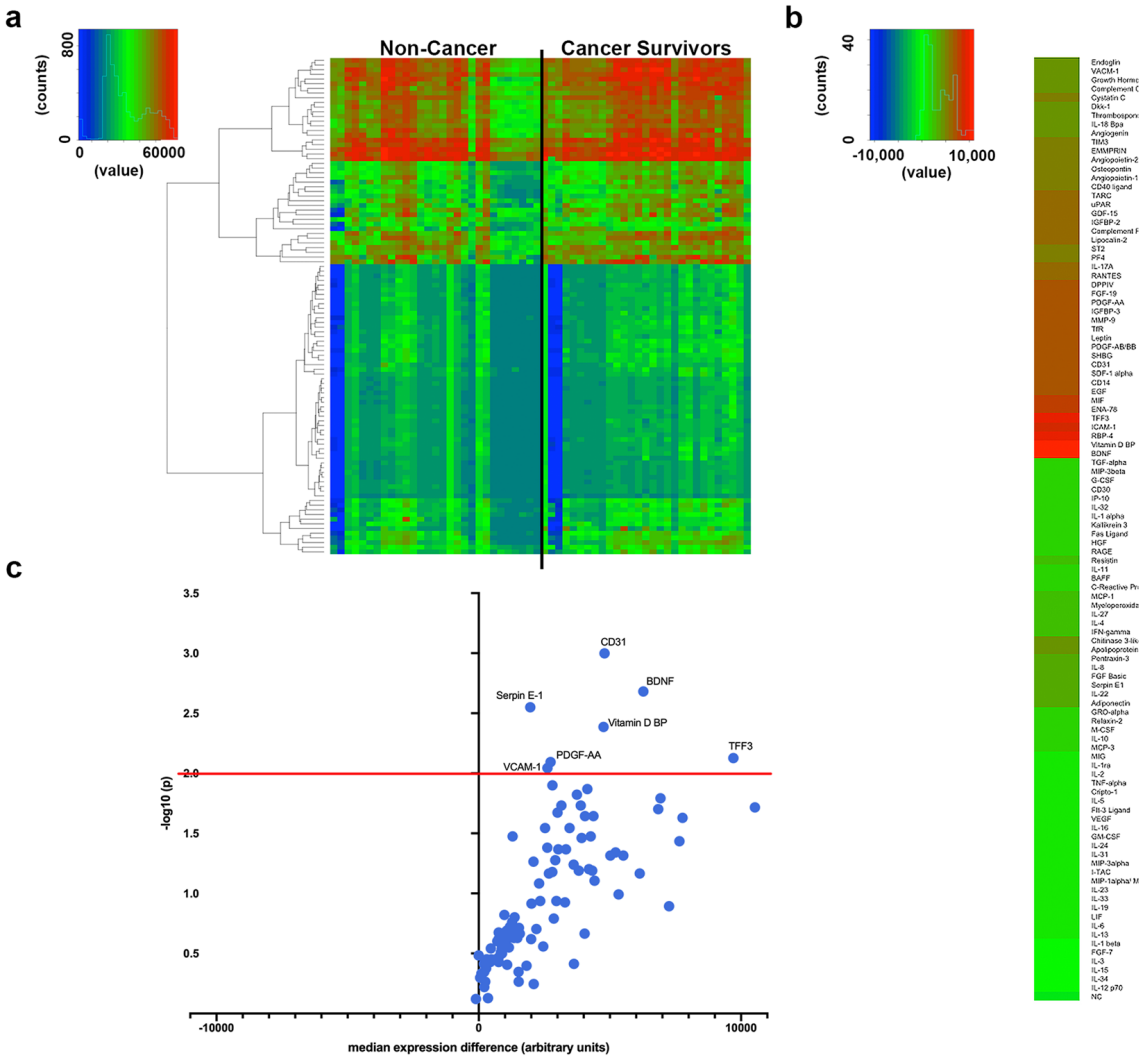
Serum cytokine expression in cancer survivors and non-cancer participants. (**a**) Heatmap depicting cytokine expression among cancer survivors and non-cancer participants. (**b**) Individual cytokine differences between cancer survivors and non-cancer participants. Higher (red) to lower (green) differences among groups. (**c**) Volcano plot depicting cytokine changes (x-axis) and p-values (statistical significance was established as *p* < 0.01; y-axis).

**Figure 3:**
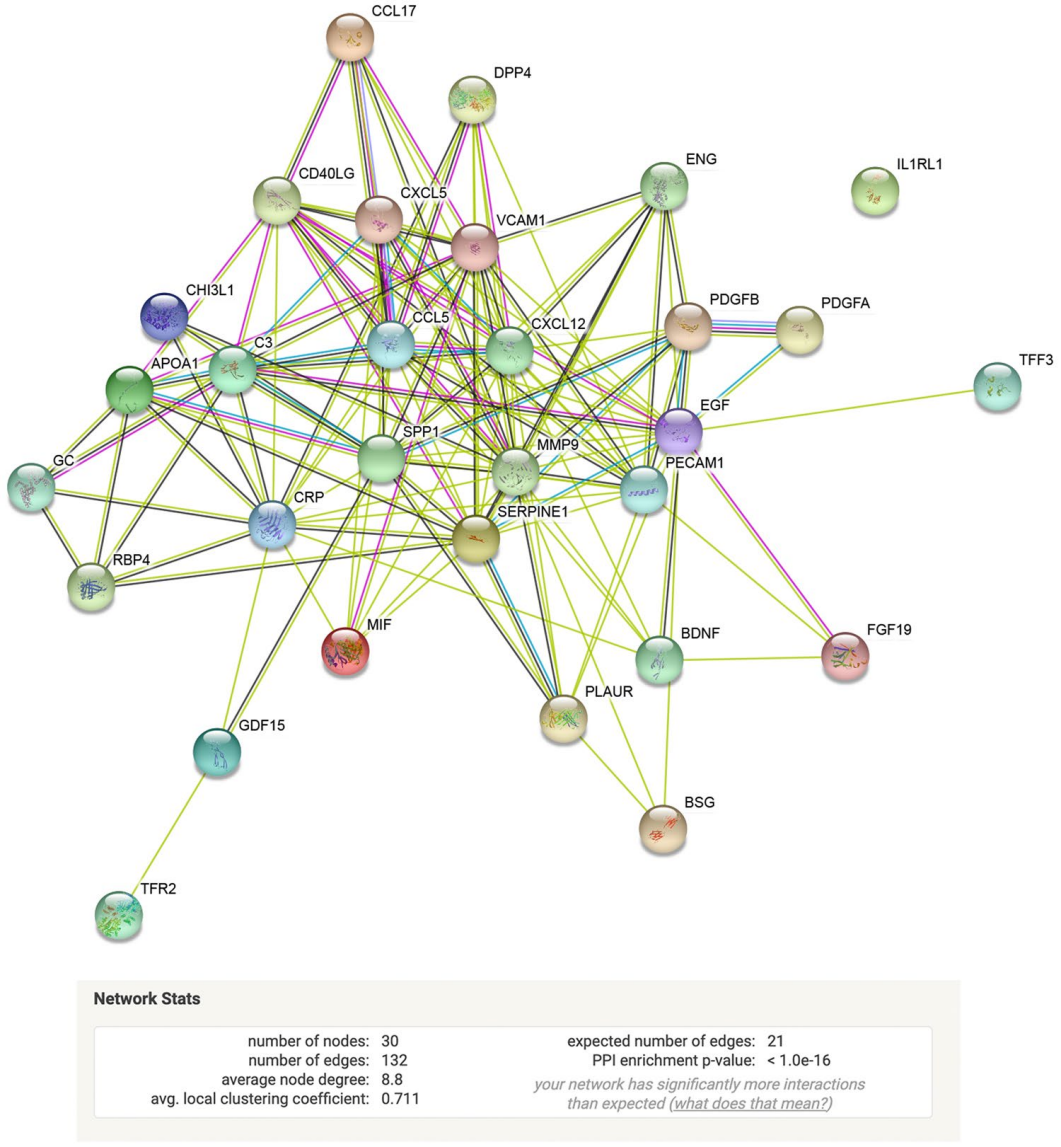
Interaction networks from significantly upregulated serum cytokines from cancer survivors. String diagram depicting molecular relationships, interactions, and pathway associations between significantly upregulated cytokines. Statistical significance for this analysis was established at *p* < 0.05.

Serum cytokines levels were analyzed from 38 cancer survivors and 30 non-cancer participants. Figure 2a shows a heatmap visualization of cytokine expression by group. Figure 2b,c shows cytokines that were significantly upregulated in the cancer survivor group when compared to non-cancer participants. The most significantly upregulated cytokines were CD31, BDNF, TFF3, Serpin E-1, Vitamin D BP, VCAM-1, PDGF-AA (Fig. 2c; *p* < 0.01). To determine possible protein-protein association networks, we data-mined the STRING V11^59^. Figure 3 depicts molecular relationships, interactions, and pathway associations between cytokines that were significantly upregulated in cancer survivors compared to non-cancer participants (threshold set at *p* < 0.05). To understand biological processes that could be modulated by these cytokines, we performed pathway enrichment analyses using STRING v11. Supplementary Table 2 depicts KEGG pathway enrichment data^60–62^ based on significantly upregulated cytokines identified in cancer survivors compared to non-cancer participants. These analyses revealed significantly enriched pathways that included leukocyte migration, PI3K-AKT, and TNF signaling pathways, cell and focal adhesion, Ras and MAPK signaling, among other pathways.

**Figure 4:**
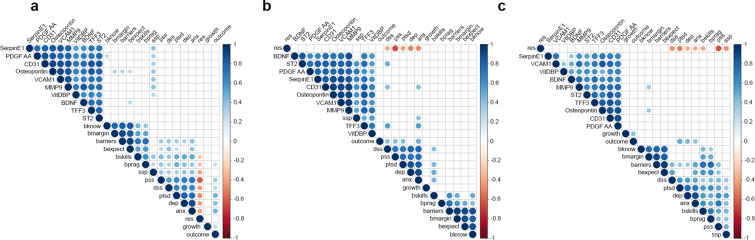
Correlation of top significantly expressed cytokines with psychosocial measurements. Top differentially expressed cytokines (by *P*-value) were subjected to Spearman correlation analyses to identify associations with psychosocial measurements among (**a**) whole cohort, (**b**) non-cancer participants, and (**c**) cancer survivors group. White squares = no significant association; Blue circles = significant positive association; Red = significant negative associations. Color intensity reflects stronger associations as determined by the correlation coefficient value (Y-axis). *P* < 0.05. *bknow*: barriers to care – knowledge and belief; *bmargin*: barriers to care – marginalization*; barriers: barriers* to care - total; *bexpect*: barriers to care – expectations; *bskills*: barriers to care – skills; *bprag*: barriers to care: pragmatics; *ssp*: social support; *pss*: perceived stress; *dss*: distress; *ptsd*: post-traumatic stress syndrome symptoms; *dep*: depressive symptomatology; *anx*: anxiety symptoms; *res*: resilience; *growth*: post-traumatic growth; *outcome*: natural disaster outcomes.

Furthermore, we explored if there was a correlation between the top ten significantly upregulated cytokines and psychosocial measures using the Spearman’s Rho correlation procedure. Figure 4 illustrates a visual representation of a correlation network with significant associations done using corrplot in RStudio 1.1.463. First, we evaluated the whole cohort and found that most cytokines had a significant positive correlation with perceived social support (Fig. 4a and Supplemental Fig. 2a). When the cohort was divided into groups, our data show that this effect was only observed in the non-cancer group (Fig. 4b,c and Supplemental Fig. 2b,c). Interestingly, in the cancer survivor group, MMP9, and Osteopontin, both known to promote disease progression^63,64^, had significant positive correlations with the marginalization scale of barriers in access to care (Fig. 4c and Supplemental Fig. 2c).

## Discussion

Our data show that Puerto Rican cancer survivors that faced HM and its aftermath suffered from a lack of access to care leading to increased health disparities among them. In fact, not only were cancer survivors presented with more barriers in accessing medical care, but our research also revealed direct relationships with anxiety, perceived stress, and post-traumatic symptomatology that were only observed in this group. Quantile regression analysis confirmed that being a cancer survivor predicted more barriers to receiving health care. This effect appears to be more pronounced early after the occurrence of the natural disaster (such as HM) and attenuated over time. Moreover, several inflammatory cytokines were found to be significantly upregulated in cancer survivors, while pathway enrichment analyses showed that these were associated with activation of tumor-promoting pathways such as those mediated by MAPK, PI3K-AKT, Ras, and TNF. Further, we uncovered a positive correlation between several cytokines and perceived social support in cancer survivors. Also, in cancer survivors, MMP9 and Osteopontin (both associated with pro-tumoral processes) levels were associated with a lack of access to care.

This study provides a new tool to identify problems that people face in the aftermath of a major hurricane. Existing scales focus mainly on exploring exposure to stressors in response to hurricanes and utilize mostly a binary response approach (1 = yes, 0 = no). They are based on surveys used after hurricanes Katrina^65,66^. For example, Ali *et al*.^67^ explored the Katrina-specific stressful exposure through an 11-item checklist that asked about having lost relatives or close people, death of a family member, or close person, having been in shelters, trapped at home or in another place. Lowe *et al*.^68^ developed the 8-item Hurricane-Related Stressors scale to explore whether participants lacked fresh water, food, medicine, or medical care, they felt their life was in danger, a family member lacked medical care and lacked knowledge of the safety of children or family members. The Natural Disaster Outcome questionnaire developed by this team expands the list of problems that people may face in response to a hurricane based on the experience of HM in PR (e.g., long lines at gas stations, traffic jams, cost of generators and maintenance, communication difficulties). Also, it can identify the severity level of each problem in a given time. Its structure is similar to the Trauma Exposure Severity Scale (TESS)^69^, which assesses stressors severity in earthquake survivors in a 24-items Likert scale (1 = not at all to 5 = extremely). However, many problems assessed by TESS do not apply to a hurricane experience and do not take into account the impact of the problems over time. Given the longitudinal nature of our study, we developed the Natural Disaster Outcome questionnaire to explore the difficulties that participants faced in the past three months, allowing us to compare the impact severity over time.

As expected, the effects of HM in PR infrastructure affected cancer survivors as well as non-cancer participants. In general, participant’s responses in most of the psychosocial measures suggest that both groups were affected equally. Although psychological distress symptoms in the total sample showed a negative and significant relationship with resilience, group analysis revealed that the association of resilience with the symptomatology of depression in the group of cancer survivors was not significant. These findings show that compared to non-cancer participants, cancer survivors report greater severity in the symptoms of depression and greater resiliency. This observation is unexpected, but it can be explained by patients’ tendency to conceal emotional distress to protect loved ones from worrying. Also, in cancer survivors, social support was positively correlated with distress. This association could be explained by Hispanic cultural values that emphasize supportive family relationships where families are prioritized before an individual’s well-being. On the other hand, the survivor’s resiliency was negatively related to barriers in access to care. Exacerbations in such barriers after the hurricane for a prolonged time can put them at risk of worsening prognoses, both physically and psychologically.

Our data identified several cytokines that have been associated with inflammatory processes, biobehavioral factors, and cancer biology^27,34,70^. For example, CD31 has been associated with angiogenic processes and cancer progression that have been shown to be promoted by chronic stress in preclinical models of cancer^28,34^. Moreover, BDNF was recently shown to be involved in cancer progression and to be modulated by chronic stress and activation of the SNS^71^. Our data also identified several signaling pathways that were enriched by cytokines found to be significantly induced. These included PI3K-Akt^72,73^, MAPK^34^, Ras, and TNF^27,34^ signaling nodes that are well-known to play key roles in cancer biology. Finally, the induction of Osteopontin and MMP9 was associated with barriers to access to care, in itself a potential source of distress. This is an important observation as these cytokines have been reported to play key roles in biobehavioral effects on cancer^63,64^. They have also been associated with metastases, cancer progression, and activation of the SNS^63,64^. These data suggest that psychosocial outcomes in the aftermath of a natural disaster could potentially play a role in cancer biology by promoting tumor-associated processes.

### Study limitations

Even though our findings provide new light of how a natural disaster can affect cancer survivors, we acknowledge that despite controlling for age and sex, our results were limited by not controlling for type of cancer, or between subjects with active disease vs. survivors, cancer stage or other comorbidities. In addition to these confounders, the limited sample size precluded our team from fully assessing the potential impact of these variables. Our analyses were also limited by utilizing self-reported data, a lack of clinical interviews to confirm psychological profiles, and not knowing the previous psychological history of participants. We acknowledge that the psychometric properties of our newly developed Natural Disaster Outcomes Questionnaire need further evaluation. Our cohort did not include a cancer survivor group that was not exposed to a natural disaster, so we cannot conclude that the biological differences seen were due to a cancer diagnosis or the hurricane. Given that multiple statistical tests were performed, setting the threshold for significance at *p* < 0.05 may yield false-positive results. Consequently, it will be imperative to confirm our observations in a larger cohort.

### Clinical implications of the study

Our data showed no significant differences between cancer survivors and non-cancer participants groups when we studied various psychosocial assessments. We observed that both groups had a high incidence of moderate depressive symptomatology and emotional distress. This is an important observation as exposure to a natural disaster not only increases the possibility of mental health symptoms but can potentially decrease the quality of life of cancer survivors. Moreover, we consider that cancer survivors can benefit from positive or appropriate social support that, in the case of this group, can help reduce negative psychosocial comorbidities and increase medical adherence. Several studies have highlighted the importance of social support networks and how this support can help overcome several common barriers to treatment, leading to better adjustment to the cancer diagnosis^74,75^. We propose that it is of paramount importance to identify factors that promote and influence resilience and well-being among Hispanic/Latinos facing a chronic or terminal illness following natural disasters. Communities and relevant groups could help by pinpointing and addressing other factors that may exacerbate cancer survivors perceived stress after a natural disaster.

### Conclusions

Our findings support changes in public policy that includes plans to ensure prompt access to treatment and specialists for cancer patients, survivors, and their caretakers, to mitigate any barriers to care in the aftermath of a hurricane. These processes will lead to better plans, care, and promote population well-being in the face of natural disasters as it is essential for stakeholders to consider the clinical and psychological needs of cancer survivors.

## Supplementary information

Supplemental Material.
