# Supplementary material for: Impact of a natural disaster on access to care and biopsychosocial outcomes among Hispanic/Latino cancer survivors
Source: Sci Rep. 2020 Jun 25;10:10376. doi: 10.1038/s41598-020-66628-z (PMC7316979; doi:10.1038/s41598-020-66628-z)
Supplement: Supplementary file 1 — Supplemental Material. [file 41598_2020_66628_MOESM1_ESM.docx]

#### Supplemental Material

#### Impact of a natural disaster on access to care and biopsychosocial outcomes among Hispanic/Latino cancer survivors

#### Mary Rodriguez-Rabassa, Ruthmarie Hernandez, Zindie Rodriguez, Claudia B. Colon-Echevarria, Lizette Maldonado, Nelmit Tollinchi, Estefania Torres-Marrero, Adnil Mulero, Daniela Albors, Jaileene Perez-Morales, Idhaliz Flores, Julie Dutil, Heather Jim, Eida M. Castro, Guillermo N. Armaiz-Pena

**Contents**

**Supplemental Data 1.** Natural Disaster Outcomes Questionnaire.

**Supplementary Table 1.** Regression coefficients for risk of barriers in access to care associated with cancer status, age, and time of recruitment after Hurricane Maria.

**Supplementary Table 2.** Identified signaling networks from significantly upregulated cytokines using KEGG database.

**Supplementary Figure 1.** Difference over time in barriers to access to care between cancer survivors and non-cancer participants.

**Supplementary Figure 2.** Correlation of top significantly expressed cytokines with psychosocial measurements.

**Supplemental Data 1. Natural Disaster Outcomes**

**Instructions:** Below is a list of situations that people may suffer after a natural disaster. Please read each situation carefully and then circle one of the numbers to the right to indicate how much you have been affected by that problem in **the past three months**.

| **In the past three months, how much were you affected by:** | **Not at all** | **A little bit** | **Moderately** | **Quite a bit** | **Extremely** |
| --- | --- | --- | --- | --- | --- |
| 1. No electricity | 0 | 1 | 2 | 3 | 4 |
| 1. Loss of home | 0 | 1 | 2 | 3 | 4 |
| 1. Loss of a loved one | 0 | 1 | 2 | 3 | 4 |
| 1. No water | 0 | 1 | 2 | 3 | 4 |
| 1. Lack of food | 0 | 1 | 2 | 3 | 4 |
| 1. Lack of food security | 0 | 1 | 2 | 3 | 4 |
| 1. Difficulty accessing treatment | 0 | 1 | 2 | 3 | 4 |
| 1. Difficulty accessing medications | 0 | 1 | 2 | 3 | 4 |
| 1. Difficulty accessing roads | 0 | 1 | 2 | 3 | 4 |
| 1. Financial issues | 0 | 1 | 2 | 3 | 4 |
| 1. Lack of home security | 0 | 1 | 2 | 3 | 4 |
| 1. Employment lay-off or reduction in labor hours | 0 | 1 | 2 | 3 | 4 |
| 1. Long lines at gas stations | 0 | 1 | 2 | 3 | 4 |
| 1. Traffic jam | 0 | 1 | 2 | 3 | 4 |
| 1. Difficulties with Internet | 0 | 1 | 2 | 3 | 4 |
| 1. Difficulties in communications | 0 | 1 | 2 | 3 | 4 |
| 1. Cost of generators acquisition | 0 | 1 | 2 | 3 | 4 |
| 1. Cost of generator maintenance (gas or diesel, oil) | 0 | 1 | 2 | 3 | 4 |
| 1. Lack of personal space | 0 | 1 | 2 | 3 | 4 |
| 1. Lack of social support | 0 | 1 | 2 | 3 | 4 |
| 1. Family separation | 0 | 1 | 2 | 3 | 4 |
| 1. Loss of vehicle | 0 | 1 | 2 | 3 | 4 |
| 1. Loss of security for my children and family | 0 | 1 | 2 | 3 | 4 |

**Consecuencias del Desastre Natural**

**Instrucciones:** Abajo tiene una lista de situaciones que las personas pueden experimentar como resultado de desastres naturales. Lea cuidadosamente cada situación y circule uno de los números a la derecha para indicar cuánto le ha molestado ese problema **en los pasados tres meses.**

| **En los pasados tres meses, cuanto le ha molestado tener:** | **No del todo** | **Un poco** | **Moderado** | **Mucho** | **Extrema-damente** |
| --- | --- | --- | --- | --- | --- |
| 1. Pérdida de electricidad | 0 | 1 | 2 | 3 | 4 |
| 1. Pérdida del hogar | 0 | 1 | 2 | 3 | 4 |
| 1. Pérdida de un ser amado | 0 | 1 | 2 | 3 | 4 |
| 1. No tener agua | 0 | 1 | 2 | 3 | 4 |
| 1. No tener alimentos | 0 | 1 | 2 | 3 | 4 |
| 1. Falta de seguridad en los alimentos | 0 | 1 | 2 | 3 | 4 |
| 1. Falta de acceso a mi tratamiento | 0 | 1 | 2 | 3 | 4 |
| 1. Falta de acceso a los medicamentos | 0 | 1 | 2 | 3 | 4 |
| 1. Falta de acceso a las carreteras | 0 | 1 | 2 | 3 | 4 |
| 1. Problemas económicos | 0 | 1 | 2 | 3 | 4 |
| 1. Falta de seguridad en el hogar | 0 | 1 | 2 | 3 | 4 |
| 1. Pérdida de empleo o reducción en la jornada laboral | 0 | 1 | 2 | 3 | 4 |
| 1. Filas largas para echar gasolina | 0 | 1 | 2 | 3 | 4 |
| 1. Tapones en la carretera | 0 | 1 | 2 | 3 | 4 |
| 1. Dificultades con el internet | 0 | 1 | 2 | 3 | 4 |
| 1. Dificultades con la comunicación | 0 | 1 | 2 | 3 | 4 |
| 1. Gasto incurrido en adquisición de generador eléctrico | 0 | 1 | 2 | 3 | 4 |
| 1. Costo de mantenimiento de generador eléctrico (gasolina o diesel, aceite) | 0 | 1 | 2 | 3 | 4 |
| 1. Falta de espacio personal | 0 | 1 | 2 | 3 | 4 |
| 1. Pérdida de apoyo social | 0 | 1 | 2 | 3 | 4 |
| 1. Separación familiar | 0 | 1 | 2 | 3 | 4 |
| 1. Pérdida de vehículo | 0 | 1 | 2 | 3 | 4 |
| 1. Pérdida de seguridad para hijos y familia | 0 | 1 | 2 | 3 | 4 |

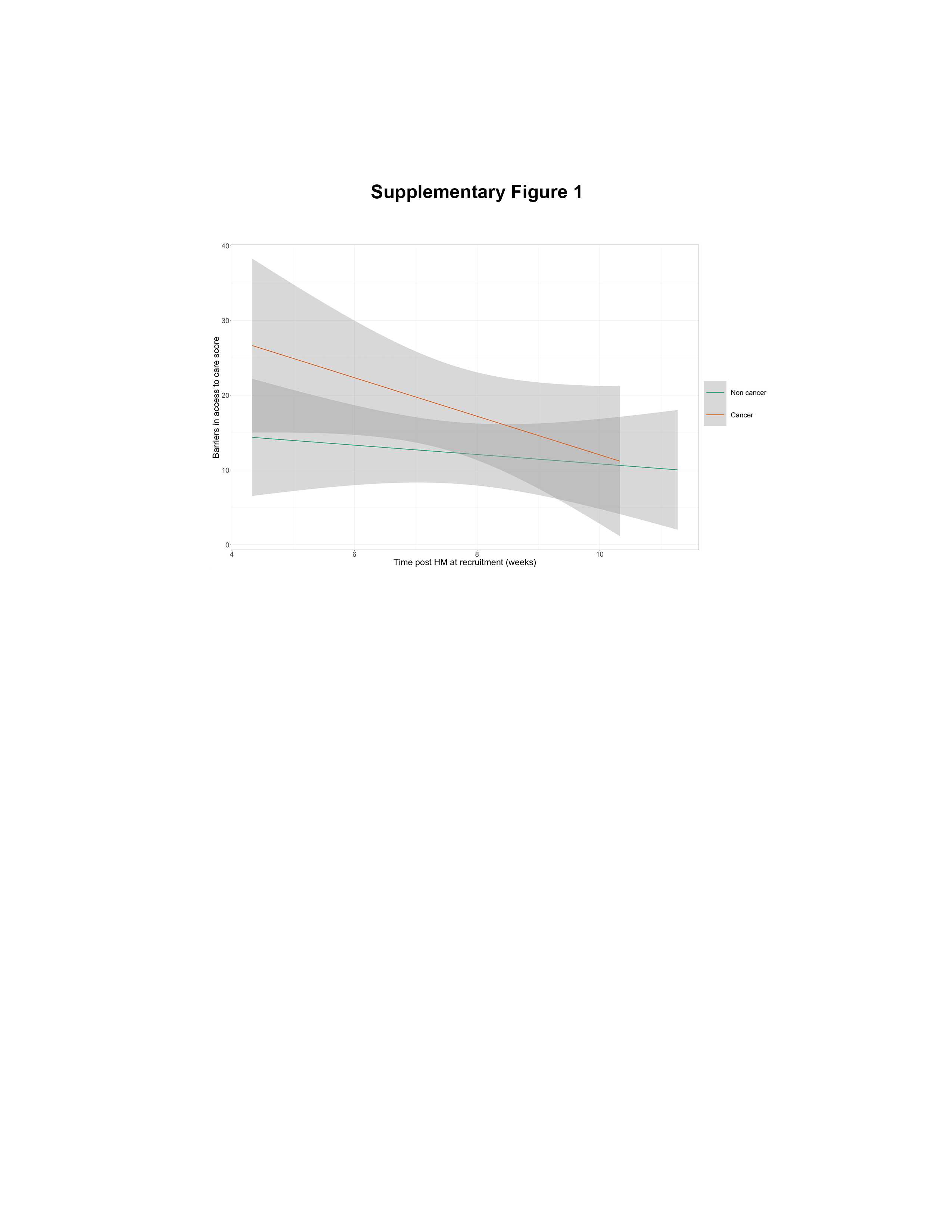


**Supplementary Figure 1. Difference over time in barriers to access to care between cancer survivors and non-cancer participants.** Here, we have investigated the relationship between time lag to recruitment after the hurricane and barriers in access to care. We observe that the difference in barriers in access to care between cancer patient and non-cancer controls is highest in the first six weeks after the hurricane and is attenuated for participants recruited more than 6 weeks after the event.


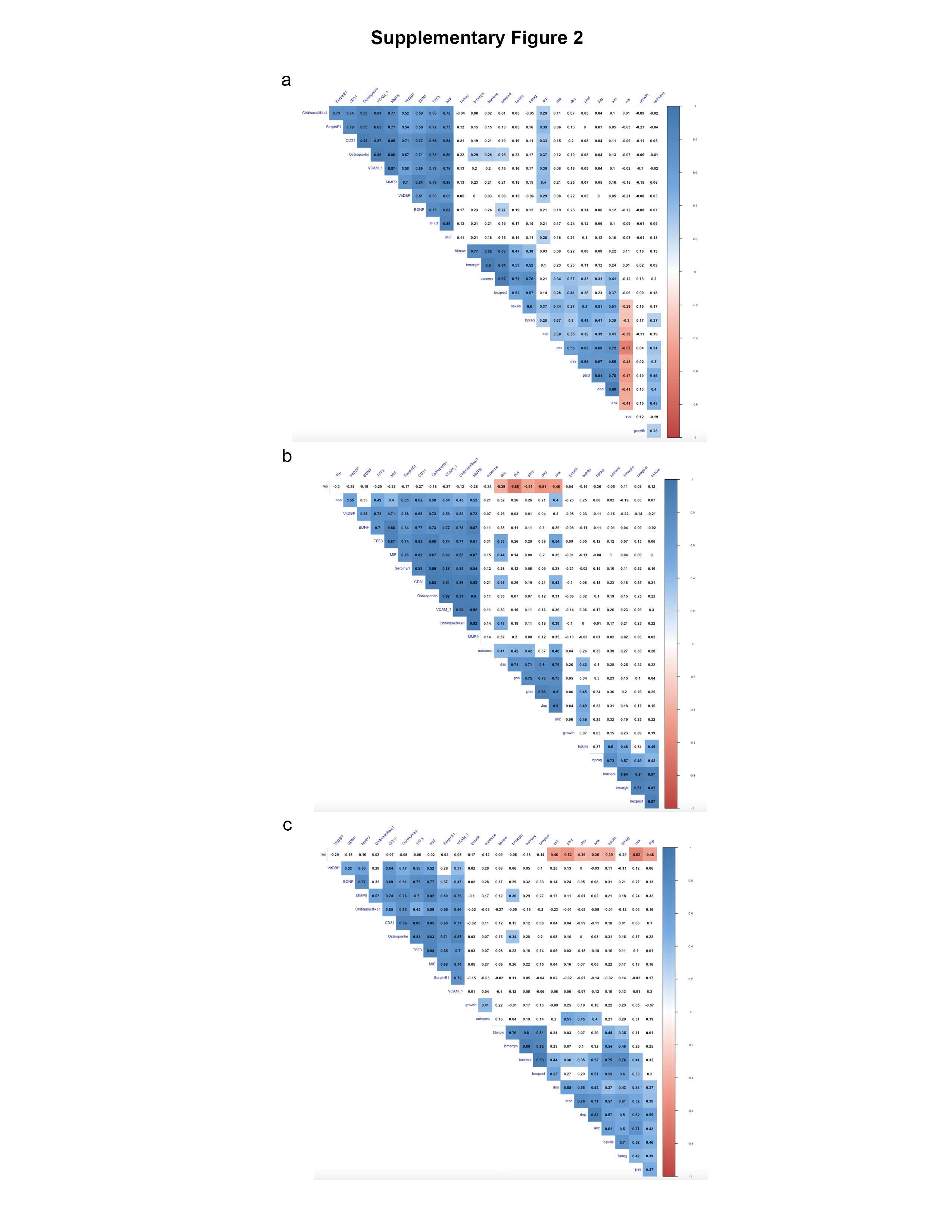


**Supplementary Figure 2. Correlation of top significantly expressed cytokines with psychosocial measurements.** Top differentially expressed cytokines (by P-value) were subjected to Spearman correlation analyses to identify associations with psychosocial measurements among (A) whole cohort, (B) non-cancer participants and (C) cancer group. R-values are shown in each square. White squares = no significant association; Blue circles = significant positive association; Red = significant negative associations. Color intensity reflects stronger associations as determined by the correlation coefficient value (Y-axis). P < 0.05. bknow: barriers to care – knowledge and belief; bmargin: barriers to care – marginalization; barriers: barriers to care - total; bexpect: barriers to care – expectations; bskills: barriers to care – skills; bprag: barriers to care: pragmatics; ssp: social support; pss: perceived stress; dss: distress; ptsd: post-traumatic stress syndrome symptoms; dep: depressive symptomatology; anx: anxiety symptoms; res: resilience; growth: post-traumatic growth; outcome: natural disaster outcomes.
